# Online nutritional disclosure of alcoholic beverages in South Africa is predominantly limited to alcohol by volume, with key nutrients omitted

**DOI:** 10.1017/S1368980026103012

**Published:** 2026-06-24

**Authors:** Fezeka Nqobile Cokile, Molatela Keneilwe Mamabolo, Khumo Molokomme, Noxolo Sibuyi, Wantonda Mukhovha, Siphiwe Ndumiso Dlamini

**Affiliations:** 1 Department of Physiology, University of the Witwatersrand Johannesburg, South Africa; 2 Faculty of Applied Sciences, Eduvos Pty Ltd, South Africa

**Keywords:** Nutritional information disclosure, Alcoholic beverages, Energy intake, Sugar content, Ingredient list

## Abstract

**Objective:**

In South Africa, as in many other countries, alcoholic beverages are not required to disclose nutritional information beyond alcohol by volume (ABV), limiting consumer awareness of energy and sugar intake. This study evaluated the extent of online nutritional information disclosure for alcoholic beverages in South Africa.

**Design:**

A cross-sectional analysis was conducted using data collected between April and September 2025. Alcoholic beverages were identified from the top five retailers by market share. A total of 3534 unique products were classified into six categories: beers, ready-to-drink beverages (RTD), red wines, white wines, sparkling wines and rosés, and spirits. Nutritional information was extracted from official brand websites, and a disclosure score was assigned based on nutrients reported. Data were analysed using chi-square and Kruskal–Wallis tests.

**Setting:**

South Africa, April-to-September 2025.

**Participants:**

N/A (product-level analysis).

**Results:**

ABV was disclosed for 83·3 % of beverages, while sugar was reported for only 33·0 % and < 3 % disclosed other nutrients or ingredient lists. Disclosure varied significantly by category (*P* < 0·0001): beers showed the widest variability (median score = 1, IQR = 1–6), white wines scored slightly higher (median = 2) and spirits the lowest (median = 0). In contrast, RTD showed consistently low disclosure, with a narrow distribution of scores (median = 1, interquartile range = 1–1).

**Conclusions:**

Most alcoholic beverages sold in South Africa predominately disclose ABV and often omit disclosure of other key nutrients. These findings underscore the need for mandatory nutritional information disclosure for alcoholic beverages to support informed consumer choices, reduce overall alcohol consumption and address alcohol-related health risks.

Non-communicable diseases (NCD) such as CVD, diabetes mellitus, cancer and chronic respiratory conditions are an increasing global health concern, accounting for about 75 % of global deaths in 2021^(1)^. This growing burden is particularly evident in sub-Saharan Africa, where the prevalence of NCD has risen by 67 % over the past two decades^(2)^. Obesity is a key driver of this trend, and in South Africa, nearly half of the adult population are overweight or obese^(3)^. Excessive alcohol consumption is recognised as an important modifiable risk factor for obesity^(4)^, with evidence showing consistent associations between excessive alcohol intake and increases in body weight. For instance, one study found that harmful alcohol consumption was associated with higher BMI and waist circumference^(5)^. Similarly, another study highlighted that heavy drinking was more consistently related to weight gain than light-to-moderate alcohol intake, suggesting a dose-dependent relationship^(6)^. Excessive alcohol consumption contributes to weight gain through two main mechanisms. First, ethanol (the active ingredient in alcoholic beverages) disrupts fat metabolism and increases appetite, leading to overeating^(7)^. Second, alcoholic beverages are often high in energy content, and consequently, this excess energy is stored by the body as fat^(6)^.

The high energy content of alcoholic beverages primarily comes from ethanol, as each gram of ethanol provides over 29 kilojoules (kJ)^(8)^. Additionally, surplus sugars and other carbohydrates left over from the fermentation process or from added sugars also contribute to the energy content^(9)^. For example, a 330 ml bottle of cider in Australia has been shown to contain as much as 724 kJ of energy^(9)^. Similarly, the inclusion of cream in many cocktails increases their energy content due to the cream’s fat composition^(9)^. Furthermore, since the body cannot store alcohol, the liver prioritises ethanol metabolism, meaning energy from alcohol is used first, while excess energy from food or drinks is stored as fat, leading to fat accumulation^(10)^. These combined effects of high energy content and metabolic prioritisation of alcohol contribute significantly to weight gain, and ultimately NCD risk^(11)^.

Given the well-established role of alcohol in obesity and NCD risk, investigating the nutritional content of alcoholic beverages is essential. Studies from developed countries have demonstrated significant variations in the nutritional content of these beverages. For example, a 2021 study from Australia found that although most packaged alcoholic beverages lacked nutrition-related information, there were significant variations in nutrient content between similar beverages^(12)^. Similarly, a study in the Czech Republic evaluated 172 beer samples and found significant energy variations, the highest energy value found was 215 kJ/100 ml, and the lowest was 75 kJ/100 ml^(13)^. Within this context, disclosing nutritional information and ingredients lists is crucial, as this transparency enables consumers to make more informed dietary choices^(14)^. Previous research has shown that providing consumers with nutritional labelling can positively influence dietary choices. For instance, one modelling study estimated that mandatory added sugar labelling for food products (alcoholic beverages were excluded) could prevent nearly one million cases of NCD^(15)^. However, in most countries, nutritional information is not available for alcoholic products. Similarly, international evidence shows strong consumer demand for transparency^(16)^. A survey conducted in the USA found that majority of alcohol consumers are in favour of mandatory labelling of alcohol content, ingredients, allergens, calories and nutrition facts on alcoholic beverages^(16)^. These findings underscore the need for improved nutritional labelling, as the lack of clear information may lead to unintentional overconsumption of energy and sugars, thereby exacerbating obesity and related NCD.

Internationally, regulatory approaches to alcohol labelling have increasingly reflected recognition of alcohol as both a psychoactive substance and a contributor to diet-related NCD. In the European Union, alcoholic beverages over 1·2 % alcohol by volume (ABV) were long exempt from mandatory nutrition labelling under Regulation (EU) No 1169/2011. Recent reforms now require energy labelling, with further nutrition and ingredient information encouraged either on-pack or via electronic tools such as QR codes^(17)^. In Australia and New Zealand, alcoholic beverages are regulated under the Food Standards Code. While full nutrition information panels remain exempt, mandatory energy labelling for packaged alcoholic products has been approved and is being phased in^(18)^.

Despite this growing body of evidence demonstrating the importance of nutritional transparency for alcoholic beverages, South Africa continues to lag in implementing policies that ensure consumers have access to such information. Many alcoholic products in the country may not disclose their nutritional information, and this may stem from a combination of regulatory and research gaps. Unlike general food products, which are governed by the Department of Health under Regulation R146 of the Foodstuffs, Cosmetics and Disinfectants Act^(19)^, alcoholic beverages occupy a regulatory grey area. Alcoholic beverages are not classified as foodstuffs and instead regulated by the Department of Agriculture, Land Reform and Rural Development under the Liquor Products Act of 1989 and related frameworks^(20)^. This regulatory separation has resulted in inconsistent labelling requirements, where nutritional declarations such as energy content, sugar levels and additives are not required for alcoholic products^(19,20)^. Furthermore, the absence of routine monitoring and surveillance of the nutritional composition of alcoholic beverages limits the availability of empirical data on population exposure to alcohol-related energy and sugars. This lack of systematic evidence constrains the ability of policymakers to quantify potential public health impacts, assess industry practices and develop or enforce evidence-informed labelling regulations, thereby slowing progress towards comprehensive alcohol labelling policies.

Beyond influencing choices between different alcoholic products, improved nutritional and ingredient disclosure may also encourage individuals to reduce or avoid alcohol consumption^(21)^. Highlighting alcohol as a source of excess energy and sugars may shift perceptions of alcohol away from being viewed as a harmless dietary component and draw attention to its role as a toxic substance with well-established adverse health effects^(22)^.

Under the Liquor Products Act, ABV is required to be disclosed on physical container labels for all alcoholic beverages sold in South Africa^(20)^. However, there is no equivalent legal requirement for ABV, or for other nutritional information, to be disclosed by online retailers or on brand websites, resulting in variable and voluntary online disclosure practices. As e-commerce expands in South Africa and consumers increasingly rely on online platforms for product information^(23)^, it is important to assess whether nutritional information on alcoholic beverages is accessible in these digital environments.

Therefore, the primary aim of this study was to investigate the proportion of alcoholic beverages that disclose nutritional information online in South Africa.

## Methods

### Study design and identification of top-performing supermarkets

This cross-sectional study included a total of 3534 unique alcoholic products. All products were identified from the online shopping websites of top-performing South African retailers, with data collected between April and September 2025. The selection of retailers was guided by the Who Owns Whom 2024 financial report on South Africa’s liquor industry^(24)^, which provided an overview of revenue trends for producers, wholesalers and the supermarkets they supply. Based on this report, the top-performing retailers in South Africa were identified as Woolworths, Makro, Pick n Pay, Checkers and Shoprite; arranged from lowest to highest revenue^(24)^.

### Selection of alcoholic beverages

**Figure 1.**
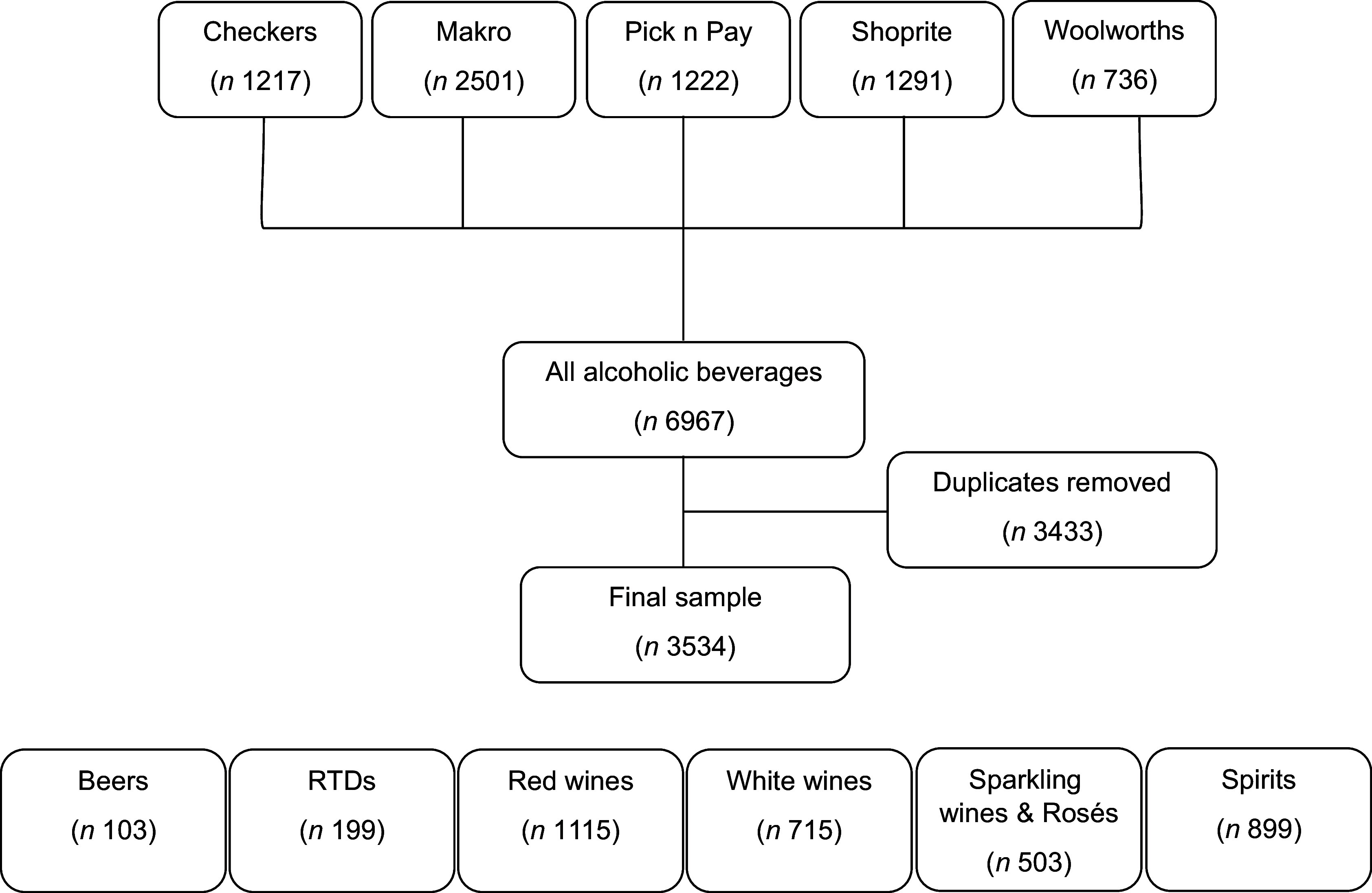
Flow diagram showing the selection of alcoholic beverages included in the analysis. Data were collected from five major South African retailers (listed in alphabetical order: Checkers, Makro, Pick n Pay, Shoprite and Woolworths), yielding to a total of 6967 alcoholic products being considered. After removal of 3433 duplicates, 3534 unique products were identified and categorised into beers (*n* 103), ready-to-drink beverages: RTD (*n* 199), red wines (*n* 1115), white wines (*n* 715), sparkling wines and rosés (*n* 503), and spirits (*n* 899).

Once the top-performing retailers were identified, a comprehensive list of all alcoholic products available online was compiled. The study included all beers, ready-to-drink beverages (RTD), red wines, white wines, sparkling wines and rosés and spirits, while solid alcoholic products (e.g. alcohol-infused chocolates) were excluded. From the official online retailer websites, an initial total of 6967 products was recorded across the five retailers: Checkers (*n* 1217), Makro (*n* 2501), Pick n Pay (*n* 1222), Shoprite (*n* 1291) and Woolworths (*n* 736), as shown in Figure 1.

Many products were listed in more than one retailer. To ensure each product was represented only once, duplicate entries were removed, resulting in the exclusion of 3433 products (Figure 1). The final sample comprised of 3534 unique alcoholic products, categorised into five main groups: Beers (*n* 103), RTD (*n* 199), red wines (*n* 1115), white wines (*n* 715), sparkling wines and rosés (*n* 503) and spirits (*n* 899) (Figure 1).

### Extraction of nutritional information and list of ingredients

All nutritional information and ingredient lists were extracted manually from official brand websites. Two trained researchers independently reviewed each product page and recorded all available nutritional information. Information displayed only in product images or photographs of labels was not captured, as these were often incomplete or inconsistently presented online. Extracted data were compared, and any discrepancies were resolved through discussion and re-examination of the original website content by either the first or last author to ensure accuracy and consistency. Nutritional information was collected per 100 ml and included total energy (kJ), protein (g), glycaemic carbohydrates (g), total sugars (g), total fat (g), saturated fat (g), dietary fibre (g) and total Na (mg), in line with the Department of Health labelling guidelines for foodstuff (R146)^(19)^. Ingredient lists were also extracted to identify the presence of added sugars and artificial sweeteners. ABV was also recorded when available online.

### Compiling a nutrient disclosure score

A nutrient disclosure score, ranging from 0 to 9, was compiled for each product to show the number of nutrients disclosed (ABV, total energy, protein, glycaemic carbohydrates, total sugar, total fat, saturated fat, dietary fibre and total sodium). A score of 0 indicated no disclosure of any nutritional information (including ABV), while a score of 9 indicated that all the above-listed nutrients were disclosed.

### Data analysis

All data analyses were conducted using R version 4.2.3. Categorical data (disclosure of the different nutrients and list of ingredients) were presented as frequencies and percentages, and differences across the beverage categories were assessed using a *χ* ^2^ test.

Normality of the continuous variables was assessed using a Shapiro–Wilk test. As the data were not normally distributed, continuous variables were presented as medians and interquartile ranges (IQR). Differences across beverage categories were assessed using a Kruskal–Wallis. When significant differences were detected, post hoc comparisons were conducted using Dunn’s test, with Bonferroni correction applied for multiple testing. A *P* value of < 0·05 was considered statistically significant.

## Results

### Disclosure of nutritional information and list of ingredients

**Figure 2.**
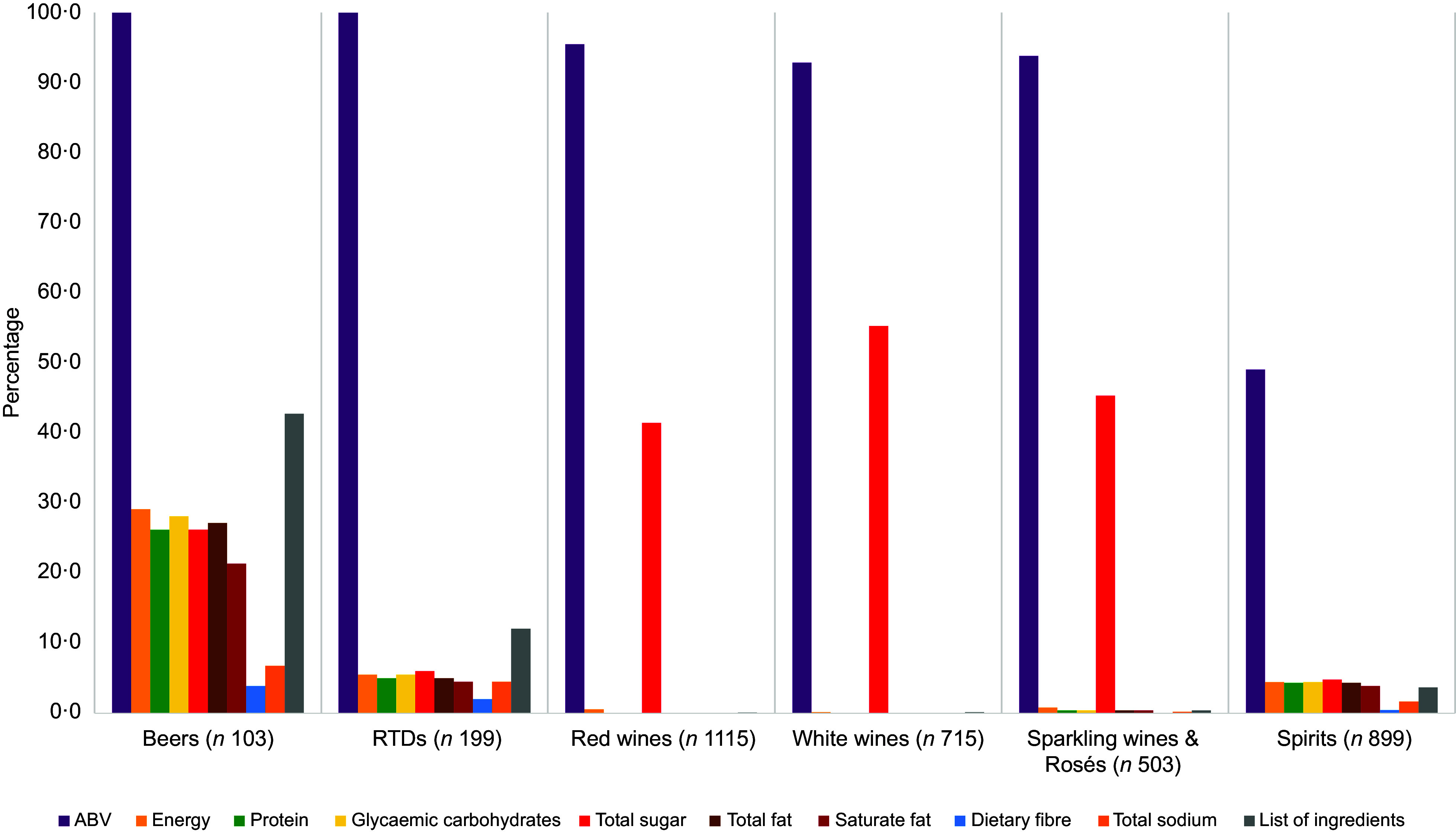
Percentages of alcoholic beverages disclosing nutritional information and/or list of ingredients on their websites. A *χ* ^2^ was used to assess differences across beverage categories, and all *P* values were < 0·0001. ABV, alcohol by volume; RTD, ready-to-drink beverages.

Figure 2 presents the proportion of all alcoholic beverage categories (beers, RTD, red wines, white wines, sparkling wines and rosés and spirits) that disclosed nutritional information on their official brand websites.

ABV and total sugar were the most frequently reported nutrients, disclosed in 83·31 % and 33·02 % of the 3534 beverages analysed, respectively. All the other nutrients were disclosed by less than 3 % of the beverages. Specifically, 2·60 % disclosed energy, 2·21 % protein, 2·32 % glycaemic carbohydrates, 2·24 % total fat, 1·92 % saturated fat, 0·41 % fibre and 0·91 % Na. Ingredient lists were only disclosed by 2·97 % of all beverages. *χ* ^2^ tests indicated strong evidence of variation in the disclosure rates of all nutrients and ingredient lists across beverage categories (all *P* < 0·0001). ABV was reported for all beers and RTD on brand websites, while reporting rates were lower for other categories: 95·52 % for red wines, 93·84 % for sparkling wines and rosés, 92·87 % for white wines and 49·05 % for spirits. Most white wines (55·24 %) disclosed sugar content, whereas disclosure rates were lower for other categories: 45·33 % for sparkling wines and rosés, 41·43 % for red wines, 26·21 % for beers, 6·03 % for RTD and 4·78 % for spirits (Figure 2).

Beers had the highest proportion of products disclosing energy content (29·13 %), followed by RTD (5·53 %), spirits (4·45 %), sparkling wines and rosés (0·80 %), red wines (0·54 %) and white wines (0·14 %). Similar patterns were observed for protein, glycaemic carbohydrates, total fat and saturated fat, across the beverage categories. Beers had the highest disclosure rates for these nutrients (26·21 %, 28·16 %, 27·18 %, 21·36 %, respectively), followed by RTD (5·03 %, 5·53 %, 5·03 %, 4·52 %, respectively), spirits (4·34 %, 4·45 %, 4·34 %, 3·89 %, respectively) and sparkling wines and rosés (all 0·40 %). None of the red wines and white wines disclosed these nutrients. The category with the highest proportion of beverages disclosing fibre and Na was beers (3·88 % and 6·80 %, respectively), followed by RTD (2·02 % and 12·06 %, respectively) and spirits (0·44 % and 1·67 %, respectively). Red wines, white wines, and sparkling wines and rosés did not disclose these nutrients. Ingredient lists were disclosed most frequently in beers (42·72 %), followed by RTD (12·06 %), spirits (3·67 %), sparkling wines and rosés (0·40 %), white wines (0·14 %) and red wines (0·09 %) (Figure 2).

### Comparing the nutrient disclosure scores

**Figure 3.**
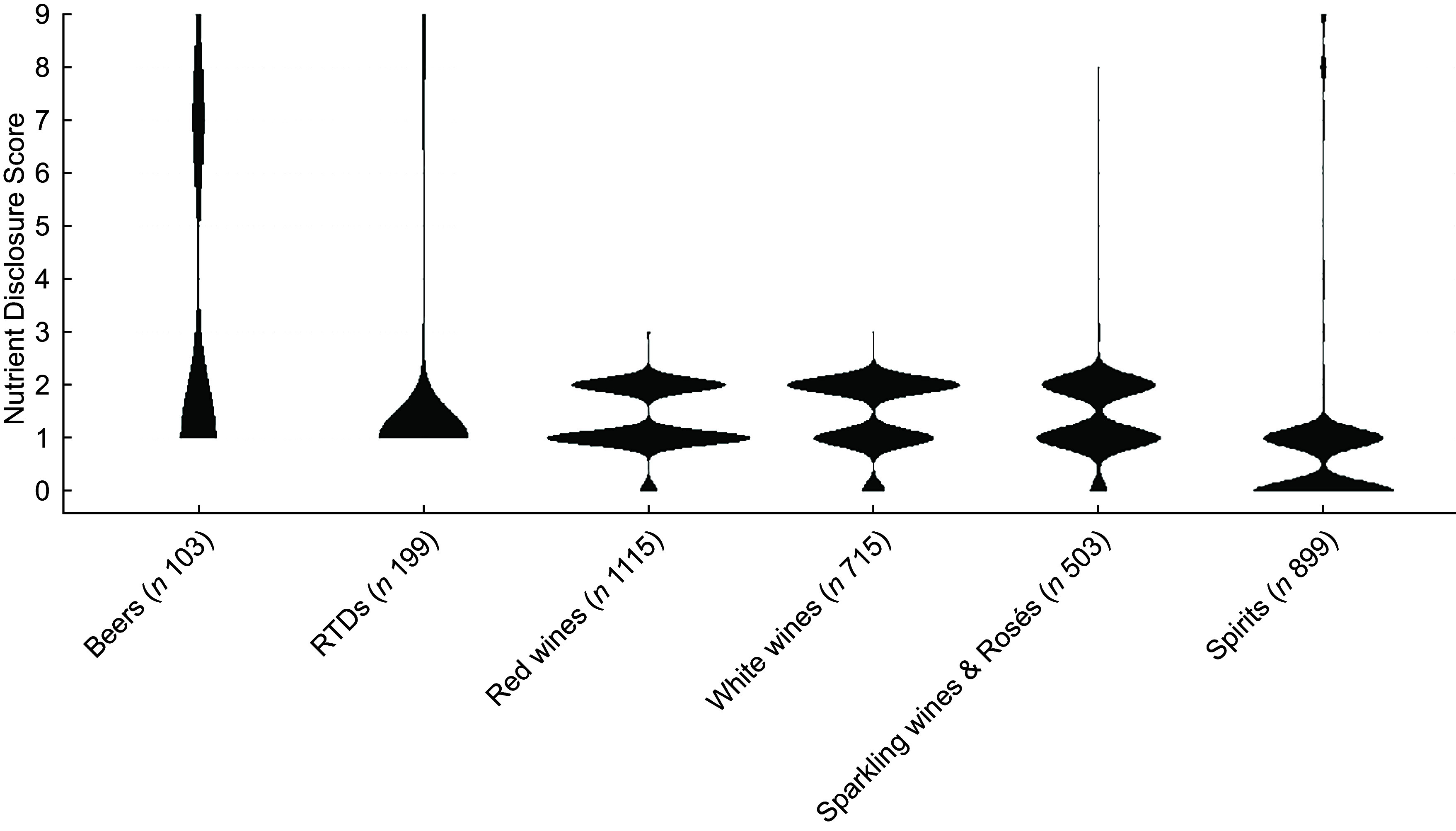
Comparison of nutrient disclosure score distributions across alcoholic beverage categories. Statistical comparisons were performed using the Kruskal–Wallis test, followed by pairwise Dunn’s tests. All Dunn’s *P* values are reported in online supplementary material, Supplemental Table S1. Overall, RTD and spirits differed significantly from all other categories (all *P* < 0·001), and red wines also differed from white wines (*P* = 0·0021). RTD, ready-to-drink beverage.

Figure 3 shows the distributions of the nutrient disclosure scores across all beverage categories. The median and IQR for the entire sample were 1(IQR: 1–2) (results not presented in the illustrations).

A Kruskal–Wallis test indicated strong evidence of differences in nutrient disclosure scores when comparing the beverage categories (*P* < 0·0001). Although the median scores for beers, RTD, red wines and sparkling wines and rosés were all 1, beers showed a wider IQR (1–6) compared with RTD (1–1), red wines (1–2) and sparkling wines and rosés (1–2). White wines had a slightly higher median score of 2 (IQR 1–2), while spirits had the lowest median score of 0 (IQR 0–1) (Figure 3). Dunn’s post hoc comparisons indicated significant differences, with both RTD and spirits differing from all other categories (All *P* < 0·001). Additionally, red wines differed from white wines (*P* = 0·0021) (see online supplementary material, Supplemental Table S1).

## Discussion

The present study is the first to investigate the proportion of alcoholic products in South Africa that disclose nutritional information and ingredient lists online. Most beverages (83 %) disclosed their ABV online, while 33 % disclosed sugar content. However, fewer than 3 % disclosed any of the other nutrients (energy, protein, glycaemic carbohydrate, fat, dietary fibre and Na). Similarly, only 3 % of beverages included a list of ingredients online, most of which (42 %) were beers. The disclosure of all nutrients and the list of ingredients varied by beverage category. Differences in disclosure were further confirmed when comparing nutrient disclosure scores, where RTD had the lowest level of disclosure.

### Disclosing alcohol by volume alone may reflect regulatory gaps

The high prevalence of ABV disclosure online likely reflects regulatory requirements. Under the Liquor Products Act 60 of 1989, ABV must appear on container labels for all alcoholic beverages sold in South Africa^(20)^. Since manufacturers are required to have this information, it is relatively easy for them to share it online. However, the legislation does not mandate disclosure on brand websites and focuses primarily on container labelling and advertising. This partly explains why, in this study, only 83 % of products disclosed ABV online. Spirits were the least likely to display ABV online, possibly due to the uniformity of alcohol content within this category. In South Africa, most spirits (such as vodka, whisky, gin, rum and brandy) are standardised to an ABV of 40 %, following regulatory amendments published on 14 March 2025 (previously, it was 43 %)^(25)^. Certain subcategories, such as potstill and vintage brandies, have a minimum ABV of 38 %, while flavoured and agave-based spirits may be as low as 35 %^(25)^. As these values are widely known and consistent across brands, manufacturers may perceive little need to display ABV online.

In contrast, the limited disclosure of other nutrients reflects gaps in current regulations. The Liquor Products Act does not mandate disclosure of energy, protein, glycaemic carbohydrate, fat, dietary fibre or Na, either on labels or online. This is likely why, in the present study, fewer than 3 % of products provided this information, highlighting that the absence of a legal requirement significantly limits voluntary disclosure.

The lack of nutritional disclosure of these other key nutrients may reflect even broader regulatory gaps. As is the case in most countries, alcoholic beverages in South Africa are not subject to regulations requiring nutritional labelling for pre-packed foodstuffs, which is under the Department of Health^(19,26)^. In South Africa, this is largely because alcohol falls under the jurisdiction of the Department of Agriculture, Land Reform and Rural Development, resulting in inconsistent labelling standards^(20)^. Consequently, this division has created a loophole that allows alcohol producers to avoid the labelling requirements imposed on other food and beverage manufacturers. For example, the South African Department of Health has recently proposed the introduction of front-of-pack nutrient labelling for unhealthy (high sugar, salt, saturated fat and/or presence of artificial sweeteners) prepacked products^(27)^. However, alcoholic beverages were excluded from these proposed regulations^(27)^. Given South Africa’s high alcohol consumption and rising obesity rates^(3,28)^, this gap poses a significant public health concern. Aligning alcohol labelling regulations with those for food products would promote consistency and fairness across the beverage industry.

### International context and opportunities for regulation

Although regulation of nutritional labelling for alcoholic beverages remains limited globally, several jurisdictions have introduced progressive policies. For instance, under Regulation (EU) 2021/2117, effective from December 2023, all wines marketed in the EU must disclose both an ingredient list and a full nutritional declaration^(17)^. Allergens must appear on the physical label, while other details may be provided electronically via QR codes^(29)^. Importantly, this requirement applies not only to EU-produced wines but also to imported products, meaning that South African wines produced after December 2023 and sold in the EU now carry QR codes linking to mandatory ingredient and nutritional information^(17)^. Ireland’s Public Health (Alcohol) Labelling Regulations 2023 will, by 2026, make it mandatory for alcoholic beverages to include comprehensive health warnings covering nutritional content, alcohol-related risks and pregnancy advisories^(30)^. Similarly, Chile has extended its front-of-package nutrient warning systems to alcoholic beverages^(31)^, while Australia and New Zealand have implemented mandatory pregnancy warnings and have recently approved mandatory energy labelling for alcoholic products, which is currently subject to a compliance transition period^(18,32)^.

It is also important to consider the form that mandatory nutritional disclosure for alcoholic beverages should take. In Australia and New Zealand, energy disclosure was prioritised over a full nutrition information panel, in part due to concerns that panels dominated by zero values for nutrients such as total fat or saturated fat could inadvertently create a ‘health halo’ effect and mislead consumers^(33)^. Given that ethanol and in some products sugars or other carbohydrates are the primary contributors to the energy content of alcoholic beverages^(8,9)^, targeted disclosure of energy or carbohydrate may therefore offer a clear and contextually meaningful approach.

In the South African context, these considerations may be particularly relevant given the commercial availability of traditional and sorghum-based beers, which can differ substantially from industrially produced alcoholic beverages in their residual carbohydrate content and overall composition^(34)^. For such products, ethanol may not be the sole contributor to energy intake, and carbohydrate levels may be higher and more variable. In these settings, broader nutritional and ingredient disclosure may have added value in supporting consumer understanding and regulatory oversight.

It is also important to recognise that nutritional information and claims on alcoholic beverages may have unintended effects. The WHO has stated that no safe level of alcohol consumption can be established and that any alcohol intake carries health risks^(35)^. In this context, claims such as ‘low sugar’ or ‘low carbohydrate’ may create ‘health halo’ effects, leading consumers to perceive certain alcoholic products as healthier options and potentially increasing consumption. Therefore, effective labelling policies should therefore prioritise clear communication of ethanol content, energy values and alcohol-related health risks and be aligned with broader alcohol control strategies aimed at reducing population-level alcohol consumption.

### Manufacturers may be concealing unfavourable nutritional profiles

It is possible that many of the beverages lacked comprehensive nutritional information because they were concealing unfavourable nutritional profiles. This was evident in the study, as ready-to-drink products, which often contain the highest sugar content, provided the least nutritional information. For instance, a study from the United Kingdom demonstrated that RTD contained extremely high levels of sugar comparable to or exceeding those found in soft drinks^(36)^. Despite these excessive sugar levels, RTD rarely displayed this information on their labels^(36)^. The omission of sugar content therefore raises the possibility that manufacturers intentionally avoid transparency to prevent negative consumer perceptions associated with high sugar intake^(37)^. This is particularly relevant because excessive sugar consumption from RTD contributes to elevated energy intake, weight gain and metabolic dysfunction^(38)^.

Our observation that beers were more likely to disclose their nutritional profiles and ingredient lists, compared with RTD, may reflect the nature of the beer production process. Beers are typically made from water, malted barley, hops and yeast^(39)^, with minimal or no added sugars or artificial sweeteners. Transparency may also be a strategic choice, as a previous study found that transparency in nutrition labelling significantly increased customer buying intentions^(40)^. Furthermore, beers generally have a lower ABV compared with wines and RTD, which may enhance their appeal to individuals seeking moderate alcohol options. In a study from Australia, alcohol consumers were shown to be increasingly choosing beers because of their lower alcohol content, as they perceive them to be healthier and less intoxicating^(41)^.

The hypothesis that manufacturers disclose nutrient information only when it enhances the perception of healthiness^(42)^ was supported by the findings of this study, particularly in the case of wines. Many wine varieties, such as dry reds, dry whites and rosés, contain minimal sugar, with only a few produced using added sugar^(43)^. In the present study, we observed that numerous wines disclosed their total sugar content, often as part of tasting notes available for download from brand websites.

The observation that spirits generally disclosed more nutrient information than wines may reflect recent international initiatives towards voluntary nutritional labelling. Leading spirit companies have introduced nutrients and ingredient information on product packaging and brand websites, a response to the growing consumer demand for transparency and aligning with global public health trends^(44)^. However, despite disclosing nutritional information online, both spirits and wines are known to generally contain relatively high alcohol content, which significantly contributes to their overall energy density, providing about twice the energy supplied by carbohydrates or proteins^(8)^. Hence, overtime, excessive alcohol consumption from these beverages contributes to fat accumulation, particularly visceral fat, which promotes metabolic disturbances such as insulin resistance and increases the risk of developing NCD^(38)^.

In addition to nutrient content, listing ingredients is equally crucial because it allows consumers to identify added sugars, preservatives, artificial sweeteners and potential allergens^(45)^. These components have been shown to influence consumer perception and behaviour^(45)^. A study from the USA demonstrated that 63 % of consumers pay closer attention to ingredient lists when shopping for food and beverages, while 30 % reported that this information affects their purchasing decisions^(46)^. Consistent with the strategy of leveraging favourable nutritional information to influence consumer perceptions of healthfulness, the present study found that beers were more likely to disclose ingredient lists compared with other alcoholic beverages. This trend may be attributed to the relatively simple and ‘healthier looking’ composition of beer^(47)^. In contrast, RTD and similar beverages often contain added sugars and other additives, which may discourage full disclosure^(36)^.

Importantly, the public health relevance of nutritional disclosure extends beyond weight management. Ethanol is a toxic substance, and alcohol consumption is causally associated with a range of adverse health outcomes, including liver disease, CVD, several cancers and neuropsychiatric disorders^(22)^. Improving the visibility of energy, sugar content and ingredients may influence how alcohol is perceived, shifting it away from being viewed as a routine dietary component towards recognition of its contribution to excess energy intake and toxic exposure^(8)^. In this context, comprehensive nutritional disclosure may support both healthier product choices and reductions in overall alcohol consumption, thereby contributing to reductions in alcohol-related harms beyond obesity.

The contrast between South Africa’s regulatory framework and recent reforms in jurisdictions such as the European Union and Australia underscores the absence of a clear policy pathway for nutritional labelling of alcoholic beverages in South Africa. While international approaches differ in scope, they share a common shift towards at least partial nutritional transparency, most notably through mandatory energy disclosure.

In the South African context, where alcohol labelling remains primarily focused on product identity and alcohol content, these international examples provide concrete reference points for incremental policy reform. Rather than adopting a full nutrition information panel identical to that used for food products, policymakers could consider targeted disclosure approaches that prioritise energy and carbohydrate content, while remaining attentive to local consumption patterns and product diversity. Such an approach may offer a pragmatic means of improving consumer information while avoiding unintended misinterpretation and would represent a step towards closer alignment with emerging international best practice.

### Study limitations and future research

A key limitation is that nutritional information was collected exclusively from online shopping and brand websites rather than from physical product labels. Although this approach was justified given the increasing use of online platforms by consumers to access product information, it is important to emphasise that any future regulatory changes would most likely mandate nutritional disclosure on physical product packaging, rather than online retail platforms. Consequently, the findings of this study may not fully reflect the level of nutritional information that would be available to consumers under a labelling-based regulatory framework.

Reliance on online data also raises concerns regarding accuracy, completeness and standardisation, as the information presented online may differ from what appears on product labels and is not subject to the same regulatory oversight. In addition, several South African wines marketed in the EU provide detailed nutritional and ingredient information primarily through QR codes linked from physical labels^(17)^. Because these QR code linked data were not consistently accessible via South African online retail platforms, they could not be included in the present analysis, potentially leading to an underestimation of disclosure among certain products.

Future research should therefore include in store assessments of physical product labels, as well as systematic inclusion of QR code linked nutritional information, to better align with how mandatory labelling regulations would likely be implemented in practice. Furthermore, laboratory analyses to verify reported nutrient values, and expansion of analyses to include all alcohol types, would provide a more comprehensive assessment of the nutritional composition and disclosure of alcoholic beverages in South Africa.

### Conclusions and recommendations

The findings of this study highlight the lack of online nutritional information disclosure on alcoholic beverages in South Africa, with most products only disclosing ABV and not reporting key nutrients such as sugar, energy and ingredient lists. This lack of transparency poses significant public health threat, particularly given the established link between excessive alcohol consumption, high sugar and energy intake and the development of obesity and NCD. The study highlights the urgent need for improved regulation of alcoholic beverages, including the implementation of mandatory nutritional labelling. Such regulations will enable consumers to make informed health choices, reduce overall alcohol intake, excessive energy intake from alcohol and mitigate obesity and NCD risk. Aligning South Africa with international best practices, such as those seen in the EU, Ireland and Australia, will provide a roadmap for improving consumer awareness and promoting healthier consumption patterns. This study not only reveals the current gaps in nutritional information disclosure but also emphasises the critical role of regulation in protecting public health. Implementing mandatory labelling has the potential to support healthier choices, reduce the burden of obesity-related NCD and improve population health outcomes in South Africa.

## Supporting information

10.1017/S1368980026103012.sm001 Cokile et al. supplementary material Cokile et al. supplementary material

## Data Availability

Data generated and all tools used in this study can be made available from the corresponding author on reasonable request.

## Supplementary material

For supplementary material accompanying this paper visit https://doi.org/10.1017/S1368980026103012
