# Supplementary material for: Online nutritional disclosure of alcoholic beverages in South Africa is predominantly limited to alcohol by volume, with key nutrients omitted
Source: Public Health Nutr. 2026 Jun 24;29(1):e144. doi: 10.1017/S1368980026103012 (PMC13434607; doi:10.1017/S1368980026103012)
Supplement: Supplementary file 1 [file S1368980026103012sup001.docx]

**Table S1: P-values from Dunnett’s post hoc comparisons of nutrient disclosure scores across alcoholic beverage categories.**

| **Nutrient disclosure score** | **Beers** | **RTDs** | **Red wines** | **Sparkling wines & Rosés** | **Spirits** |
| --- | --- | --- | --- | --- | --- |
| **RTDs** | 0.0005* |  |  |  |  |
| **Red wines** | 1.0000 | 0.0000* |  |  |  |
| **Sparkling wines & Rosés** | 1.0000 | 0.0000* | 1.0000 |  |  |
| **Spirits** | 0.0000* | 0.0000* | 0.0000* | 0.0000* |  |
| **White wines** | 1.0000 | 0.0000* | 0.0021* | 0.1551 | 0.0000* |

RTDs: Ready-to-drink beverages. *Statistically significant.
